# Crystal structure of 1-(8-meth­oxy-2*H*-chromen-3-yl)ethanone

**DOI:** 10.1107/S1600536814016808

**Published:** 2014-08-01

**Authors:** Dongsoo Koh

**Affiliations:** a Department of Applied Chemistry, Dongduk Women’s University, 23-1 Wolkok-dong, Sungbuk-ku, Seoul, 136-714, Republic of Korea

**Keywords:** crystal structure, hydrogen bonding, di­hydro­pyran ring, chromenes

## Abstract

In the structure of the title compound, C_12_H_12_O_3_, the di­hydro­pyran ring is fused with the benzene ring. The di­hydro­pyran ring is in a half-chair conformation, with the ring O and methyl­ene C atoms positioned 1.367 (3) and 1.504 (4) Å, respectively, on either side of the mean plane formed by the other four atoms. The meth­oxy group is coplanar with the benzene ring to which it is connected [C_b_—Cb—O_m_—C_m_ torsion angle = −0.2 (4)°; b = benzene and m = meth­oxy], and similarly the aldehyde is coplanar with respect to the double bond of the di­hydro­pyran ring [C_dh_—C_dh_—C_a_—O_a_ = −178.1 (3)°; dh = di­hydro­pyran and a = aldehyde]. In the crystal, mol­ecules are linked by weak meth­yl–meth­oxy C—H⋯O hydrogen bonds into supra­molecular chains along the *a-*axis direction.

## Related literature   

For the synthesis and biological properties of chromene derivatives, see: Choi *et al.* (2014 ▶); Mun *et al.* (2012 ▶); Yoon *et al.* (2012 ▶). For the chromene group in natural products, see: Starks *et al.* (2014 ▶); Escandón-Rivera *et al.* (2012 ▶). For related structures, see: Yan & Zhang (2013 ▶): Yusufzai *et al.* (2012 ▶).
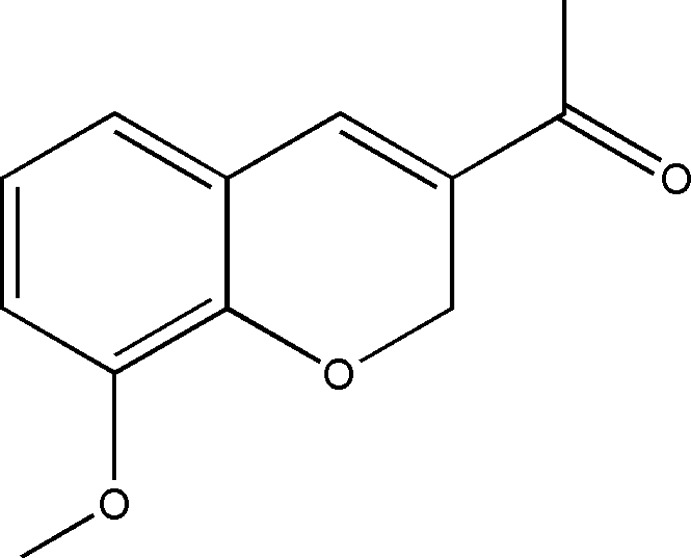


## Experimental   

### Crystal data  

C_12_H_12_O_3_
*M* *_r_* = 204.22
Orthorhombic, 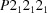

*a* = 5.1000 (4) Å
*b* = 12.7455 (9) Å
*c* = 15.130 (1) Å
*V* = 983.48 (12) Å^3^
*Z* = 4
Mo *K*α radiation
μ = 0.10 mm^−1^
*T* = 173 K
0.26 × 0.20 × 0.04 mm

### Data collection  

Bruker SMART CCD area-detector diffractometer
7256 measured reflections
2439 independent reflections
1943 reflections with *I* > 2σ(*I*)
*R*_int_ = 0.029

### Refinement  

*R*[*F* ^2^ > 2σ(*F* ^2^)] = 0.041
*wR*(*F* ^2^) = 0.148
*S* = 1.15
2439 reflections
138 parameters
H-atom parameters constrained
Δρ_max_ = 0.30 e Å^−3^
Δρ_min_ = −0.36 e Å^−3^

### 

Data collection: *SMART* (Bruker, 2000 ▶); cell refinement: *SAINT* (Bruker, 2000 ▶); data reduction: *SAINT*; program(s) used to solve structure: *SHELXS97* (Sheldrick, 2008 ▶); program(s) used to refine structure: *SHELXL97* (Sheldrick, 2008 ▶); molecular graphics: *SHELXTL* (Sheldrick, 2008 ▶); software used to prepare material for publication: *SHELXTL*.

## Supplementary Material

Crystal structure: contains datablock(s) I, New_Global_Publ_Block, global. DOI: 10.1107/S1600536814016808/tk5330sup1.cif

Structure factors: contains datablock(s) I. DOI: 10.1107/S1600536814016808/tk5330Isup2.hkl

Supporting information file. DOI: 10.1107/S1600536814016808/tk5330Isup3.cml

**Fig. 1.**
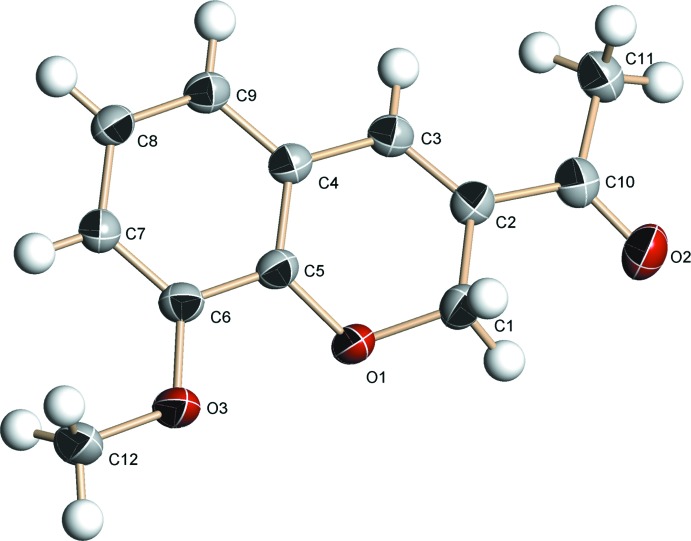
The molecular structure of the title compound, showing the atom labelling scheme and displacement ellipsoids drawn at the 50% probability level.

. DOI: 10.1107/S1600536814016808/tk5330fig1.tif
The mol­ecular structure of the title compound, showing the atom labelling scheme and displacement ellipsoids drawn at the 50% probability level.

**Fig. 2.**
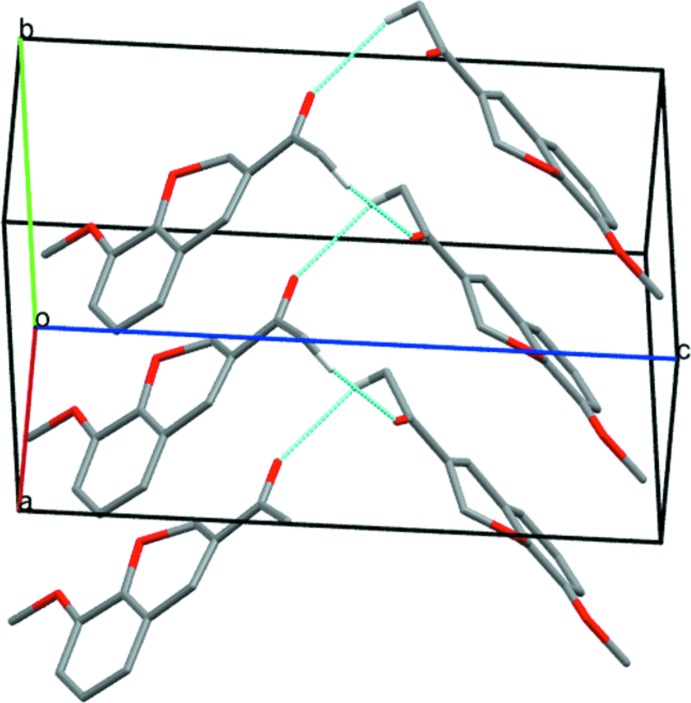
Part of the crystal structure with weak intermolecular C—H···O hydrogen bonds shown as dashed lines.

. DOI: 10.1107/S1600536814016808/tk5330fig2.tif
Part of the crystal structure with weak inter­molecular C—H⋯O hydrogen bonds shown as dashed lines.

CCDC reference: 1015076

Additional supporting information:  crystallographic information; 3D view; checkCIF report

## Figures and Tables

**Table 1:** Hydrogen-bond geometry (Å, °)

*D*—H⋯*A*	*D*—H	H⋯*A*	*D*⋯*A*	*D*—H⋯*A*
C11—H11*A*⋯O2^i^	0.98	2.56	3.429 (4)	148

Symmetry code: (i) 
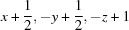
.

## supplementary crystallographic information

### S2.1. Synthesis and crystallization

To a solution of 2-hy­droxy-3-meth­oxy-benzaldehyde (750 mg, 5 mmol) in 1,4-dioxane (15 ml) was added excess amount of methyl vinyl ketone (0.7 mL, 8 mmol) and potassium carbonate (700 mg, 5 mmol) at room temperature. The reaction mixture was refluxed for 12 h and TLC showed no evidence for the starting material. After cooling to room temperature, the mixture was poured into iced water (40 ml) and extracted with methyl­ene chloride (3 x 20 ml) and the combined organic layers were dried under MgSO_4_. Filtration, evaporation of filtrate gave residue which was purified by flash chromatography to give the titled compound (22%). Recrystallization from its ethanol solution gave crystals (M.pt: 375–376 K).

### S2.2. Refinement

The H atoms were placed in calculated positions and refined as riding with C—H = 0.95 Å, and with *U*_iso_(H) = 1.2*U*_eq_(C).

### S3. Results and discussion

Chromenes have been shown to be potential pharmaceuticals which show anti-inflammatory (Choi *et al.*, 2014) and anti­cancer (Mun *et al.*, 2012) activities. Especially, the 2*H*-chromene skeleton is a core structure of oxygen heterocycles in many natural products having versatile biological activities (Starks *et al.*, 2014; Escandón-Rivera *et al.*, 2012). In continuation of our research inter­est to develop novel chromene derivatives (Yoon *et al.*, 2012), the title compound was synthesized and its crystal structure was determined (Fig. 1). In the chromene compound, the di­hydro­pyran ring is fused with the benzene ring and is in a half-chair conformation with atoms C1 and O1 positioned 1.367 (3) and 1.504 (4) Å, respectively, on either side of the mean plane formed by the other four atoms (C2/C3/C4/C5). In the crystal, weak C—H—-O hydrogen bonds link molecules along [100] (Fig. 2). Examples of structures of chromene compounds have been published (Yan *et al.*, 2013; Yusufzai *et al.*, 2012).

### Crystal data

**Table:**

C_12_H_12_O_3_	*F*(000) = 432
*M**_r_* = 204.22	*D*_x_ = 1.379 Mg m^−^^3^
Orthorhombic, *P*2_1_2_1_2_1_	Mo *K*α radiation, λ = 0.71073 Å
Hall symbol: P 2ac 2ab	Cell parameters from 4995 reflections
*a* = 5.1000 (4) Å	θ = 2.7–28.3°
*b* = 12.7455 (9) Å	µ = 0.10 mm^−^^1^
*c* = 15.130 (1) Å	*T* = 173 K
*V* = 983.48 (12) Å^3^	Block, white
*Z* = 4	0.26 × 0.20 × 0.04 mm

### Data collection

**Table:**

Bruker SMART CCD area-detector diffractometer	1943 reflections with *I* > 2σ(*I*)
Radiation source: fine-focus sealed tube	*R*_int_ = 0.029
Graphite monochromator	θ_max_ = 28.3°, θ_min_ = 2.1°
φ and ω scans	*h* = −4→6
7256 measured reflections	*k* = −16→16
2439 independent reflections	*l* = −20→19

### Refinement

**Table:**

Refinement on *F*^2^	Primary atom site location: structure-invariant direct methods
Least-squares matrix: full	Secondary atom site location: difference Fourier map
*R*[*F*^2^ > 2σ(*F*^2^)] = 0.041	Hydrogen site location: inferred from neighbouring sites
*wR*(*F*^2^) = 0.148	H-atom parameters constrained
*S* = 1.15	*w* = 1/[σ^2^(*F*_o_^2^) + (0.0443*P*)^2^ + 0.8069*P*] where *P* = (*F*_o_^2^ + 2*F*_c_^2^)/3
2439 reflections	(Δ/σ)_max_ < 0.001
138 parameters	Δρ_max_ = 0.30 e Å^−^^3^
0 restraints	Δρ_min_ = −0.36 e Å^−^^3^

### Special details

**Table:**

Geometry. All e.s.d.'s (except the e.s.d. in the dihedral angle between two l.s. planes) are estimated using the full covariance matrix. The cell e.s.d.'s are taken into account individually in the estimation of e.s.d.'s in distances, angles and torsion angles; correlations between e.s.d.'s in cell parameters are only used when they are defined by crystal symmetry. An approximate (isotropic) treatment of cell e.s.d.'s is used for estimating e.s.d.'s involving l.s. planes.
Refinement. Refinement of *F*^2^ against ALL reflections. The weighted *R*-factor *wR* and goodness of fit *S* are based on *F*^2^, conventional *R*-factors *R* are based on *F*, with *F* set to zero for negative *F*^2^. The threshold expression of *F*^2^ > σ(*F*^2^) is used only for calculating *R*-factors(gt) *etc*. and is not relevant to the choice of reflections for refinement. *R*-factors based on *F*^2^ are statistically about twice as large as those based on *F*, and *R*- factors based on ALL data will be even larger.

### Fractional atomic coordinates and isotropic or equivalent isotropic displacement parameters (Å^2^)

**Table:**

	*x*	*y*	*z*	*U*_iso_*/*U*_eq_	
O1	0.2139 (4)	0.13132 (13)	0.78457 (12)	0.0369 (5)	
C1	0.0935 (6)	0.1378 (2)	0.69905 (18)	0.0382 (6)	
H1A	−0.0596	0.0900	0.6976	0.046*	
H1B	0.2202	0.1130	0.6541	0.046*	
C2	0.0045 (5)	0.2465 (2)	0.67470 (17)	0.0317 (5)	
C3	0.1318 (5)	0.32861 (19)	0.70942 (16)	0.0315 (5)	
H3	0.0841	0.3977	0.6921	0.038*	
C4	0.3416 (5)	0.31384 (19)	0.77314 (16)	0.0290 (5)	
C5	0.3744 (5)	0.21278 (18)	0.80743 (16)	0.0304 (5)	
C6	0.5662 (5)	0.19364 (19)	0.87130 (17)	0.0323 (6)	
C7	0.7236 (6)	0.27498 (19)	0.90030 (17)	0.0327 (5)	
H7	0.8543	0.2621	0.9437	0.039*	
C8	0.6916 (6)	0.3759 (2)	0.86613 (17)	0.0335 (6)	
H8	0.8013	0.4314	0.8860	0.040*	
C9	0.5011 (5)	0.39520 (19)	0.80357 (17)	0.0320 (5)	
H9	0.4783	0.4642	0.7811	0.038*	
C10	−0.2116 (5)	0.2531 (2)	0.61005 (18)	0.0357 (6)	
O2	−0.3175 (4)	0.17219 (16)	0.58488 (14)	0.0471 (5)	
C11	−0.2923 (6)	0.3576 (2)	0.57362 (18)	0.0414 (7)	
H11A	−0.1666	0.3797	0.5283	0.062*	
H11B	−0.2956	0.4095	0.6214	0.062*	
H11C	−0.4673	0.3519	0.5473	0.062*	
O3	0.5790 (4)	0.09230 (15)	0.90143 (14)	0.0430 (5)	
C12	0.7723 (6)	0.0714 (2)	0.9676 (2)	0.0435 (7)	
H12A	0.9471	0.0861	0.9437	0.065*	
H12B	0.7620	−0.0025	0.9853	0.065*	
H12C	0.7403	0.1162	1.0191	0.065*	

### Atomic displacement parameters (Å^2^)

**Table:**

	*U*^11^	*U*^22^	*U*^33^	*U*^12^	*U*^13^	*U*^23^
O1	0.0406 (10)	0.0278 (9)	0.0424 (10)	−0.0065 (8)	−0.0104 (9)	0.0029 (7)
C1	0.0443 (15)	0.0297 (12)	0.0405 (13)	−0.0008 (12)	−0.0109 (13)	−0.0034 (11)
C2	0.0296 (12)	0.0307 (12)	0.0350 (12)	0.0021 (10)	0.0010 (10)	−0.0026 (10)
C3	0.0318 (13)	0.0285 (12)	0.0341 (12)	0.0045 (10)	0.0022 (11)	−0.0001 (10)
C4	0.0286 (12)	0.0268 (11)	0.0316 (12)	0.0013 (10)	0.0034 (10)	−0.0018 (9)
C5	0.0336 (13)	0.0257 (11)	0.0318 (12)	−0.0022 (10)	0.0013 (11)	−0.0016 (9)
C6	0.0347 (13)	0.0272 (12)	0.0349 (12)	0.0009 (10)	−0.0002 (11)	0.0018 (10)
C7	0.0335 (13)	0.0334 (13)	0.0311 (12)	−0.0017 (10)	−0.0015 (11)	−0.0003 (10)
C8	0.0379 (14)	0.0285 (12)	0.0342 (12)	−0.0044 (11)	0.0000 (11)	−0.0013 (10)
C9	0.0348 (13)	0.0261 (11)	0.0351 (12)	0.0023 (10)	0.0034 (11)	−0.0016 (10)
C10	0.0340 (13)	0.0400 (14)	0.0330 (12)	0.0047 (12)	0.0014 (11)	−0.0041 (11)
O2	0.0446 (12)	0.0441 (12)	0.0527 (12)	−0.0028 (10)	−0.0110 (10)	−0.0106 (9)
C11	0.0437 (16)	0.0451 (15)	0.0354 (13)	0.0064 (14)	−0.0037 (12)	0.0011 (11)
O3	0.0498 (12)	0.0285 (9)	0.0508 (11)	−0.0036 (9)	−0.0161 (10)	0.0082 (8)
C12	0.0464 (17)	0.0345 (14)	0.0497 (16)	0.0006 (13)	−0.0131 (14)	0.0096 (12)

### Geometric parameters (Å, º)

**Table:**

O1—C5	1.366 (3)	C7—C8	1.396 (3)
O1—C1	1.435 (3)	C7—H7	0.9500
C1—C2	1.504 (4)	C8—C9	1.378 (4)
C1—H1A	0.9900	C8—H8	0.9500
C1—H1B	0.9900	C9—H9	0.9500
C2—C3	1.339 (4)	C10—O2	1.224 (3)
C2—C10	1.476 (4)	C10—C11	1.499 (4)
C3—C4	1.452 (4)	C11—H11A	0.9800
C3—H3	0.9500	C11—H11B	0.9800
C4—C9	1.396 (3)	C11—H11C	0.9800
C4—C5	1.399 (3)	O3—C12	1.430 (3)
C5—C6	1.397 (3)	C12—H12A	0.9800
C6—O3	1.371 (3)	C12—H12B	0.9800
C6—C7	1.383 (4)	C12—H12C	0.9800
			
C5—O1—C1	116.2 (2)	C8—C7—H7	119.8
O1—C1—C2	113.8 (2)	C9—C8—C7	120.1 (2)
O1—C1—H1A	108.8	C9—C8—H8	120.0
C2—C1—H1A	108.8	C7—C8—H8	120.0
O1—C1—H1B	108.8	C8—C9—C4	120.3 (2)
C2—C1—H1B	108.8	C8—C9—H9	119.8
H1A—C1—H1B	107.7	C4—C9—H9	119.8
C3—C2—C10	125.3 (2)	O2—C10—C2	119.2 (2)
C3—C2—C1	118.5 (2)	O2—C10—C11	120.8 (2)
C10—C2—C1	116.1 (2)	C2—C10—C11	119.9 (2)
C2—C3—C4	121.1 (2)	C10—C11—H11A	109.5
C2—C3—H3	119.5	C10—C11—H11B	109.5
C4—C3—H3	119.5	H11A—C11—H11B	109.5
C9—C4—C5	119.5 (2)	C10—C11—H11C	109.5
C9—C4—C3	123.5 (2)	H11A—C11—H11C	109.5
C5—C4—C3	117.0 (2)	H11B—C11—H11C	109.5
O1—C5—C6	117.5 (2)	C6—O3—C12	116.2 (2)
O1—C5—C4	122.3 (2)	O3—C12—H12A	109.5
C6—C5—C4	120.1 (2)	O3—C12—H12B	109.5
O3—C6—C7	125.0 (2)	H12A—C12—H12B	109.5
O3—C6—C5	115.3 (2)	O3—C12—H12C	109.5
C7—C6—C5	119.7 (2)	H12A—C12—H12C	109.5
C6—C7—C8	120.4 (2)	H12B—C12—H12C	109.5
C6—C7—H7	119.8		
			
C5—O1—C1—C2	−39.4 (3)	O1—C5—C6—C7	−176.4 (2)
O1—C1—C2—C3	28.1 (4)	C4—C5—C6—C7	−0.1 (4)
O1—C1—C2—C10	−154.7 (2)	O3—C6—C7—C8	−179.0 (3)
C10—C2—C3—C4	179.7 (2)	C5—C6—C7—C8	0.0 (4)
C1—C2—C3—C4	−3.5 (4)	C6—C7—C8—C9	0.5 (4)
C2—C3—C4—C9	172.2 (2)	C7—C8—C9—C4	−0.9 (4)
C2—C3—C4—C5	−10.5 (4)	C5—C4—C9—C8	0.8 (4)
C1—O1—C5—C6	−156.5 (2)	C3—C4—C9—C8	178.1 (2)
C1—O1—C5—C4	27.3 (3)	C3—C2—C10—O2	−178.1 (3)
C9—C4—C5—O1	175.8 (2)	C1—C2—C10—O2	5.0 (4)
C3—C4—C5—O1	−1.7 (4)	C3—C2—C10—C11	4.2 (4)
C9—C4—C5—C6	−0.3 (4)	C1—C2—C10—C11	−172.7 (2)
C3—C4—C5—C6	−177.7 (2)	C7—C6—O3—C12	−0.2 (4)
O1—C5—C6—O3	2.8 (3)	C5—C6—O3—C12	−179.3 (2)
C4—C5—C6—O3	179.0 (2)		

### Hydrogen-bond geometry (Å, º)

**Table:**

*D*—H···*A*	*D*—H	H···*A*	*D*···*A*	*D*—H···*A*
C11—H11*A*···O2^i^	0.98	2.56	3.429 (4)	148

Symmetry code: (i) *x*+1/2, −*y*+1/2, −*z*+1.
