# Supplementary figures and images for: Crystal structure of 1-(8-meth­oxy-2H-chromen-3-yl)ethanone
Source: Acta Crystallogr Sect E Struct Rep Online. 2014 Aug 1;70(Pt 9):o936–7. doi: 10.1107/S1600536814016808 (PMC4186109; doi:10.1107/S1600536814016808)

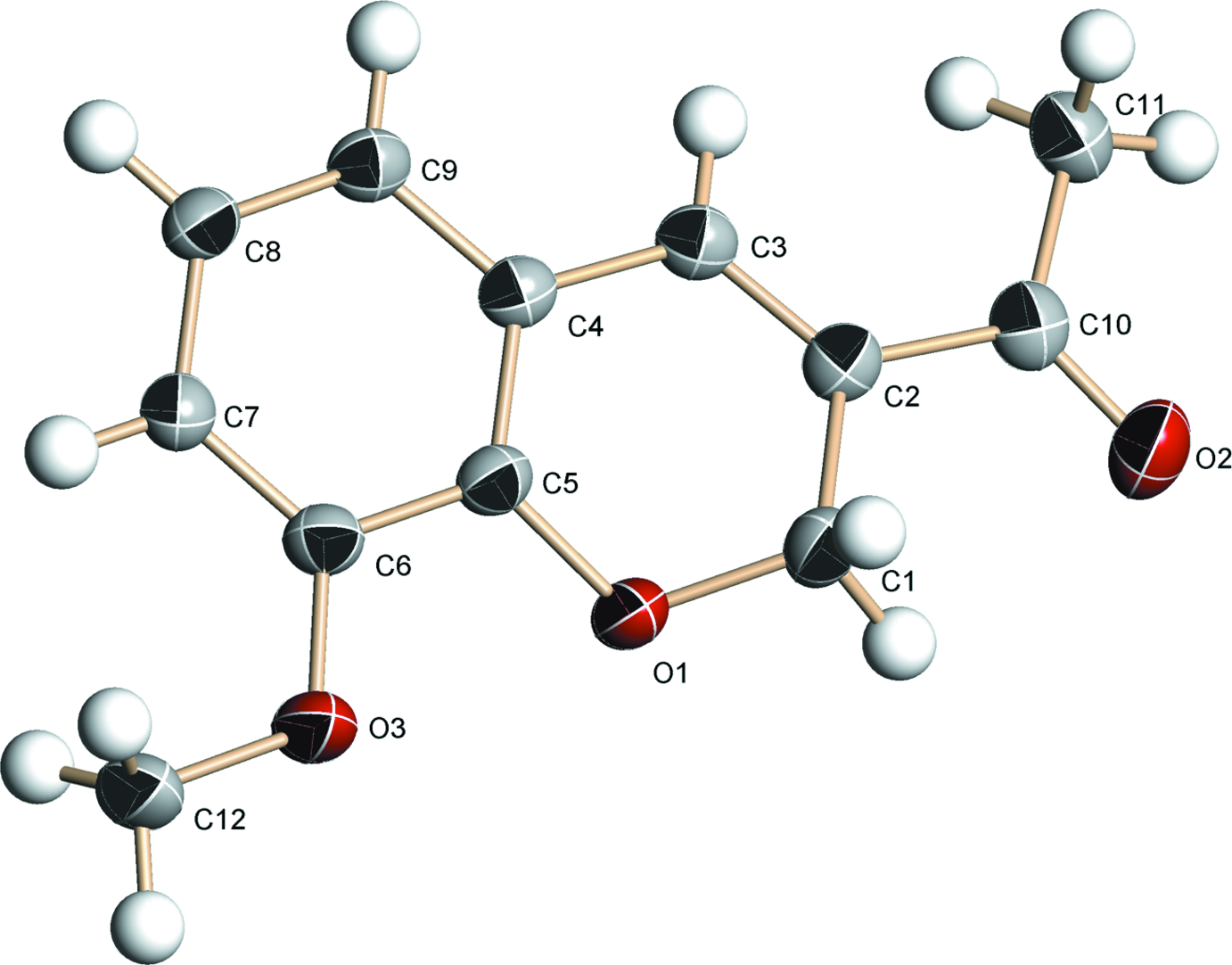

Supplement: Supplementary file 4 [file e-70-0o936-fig1.tif]

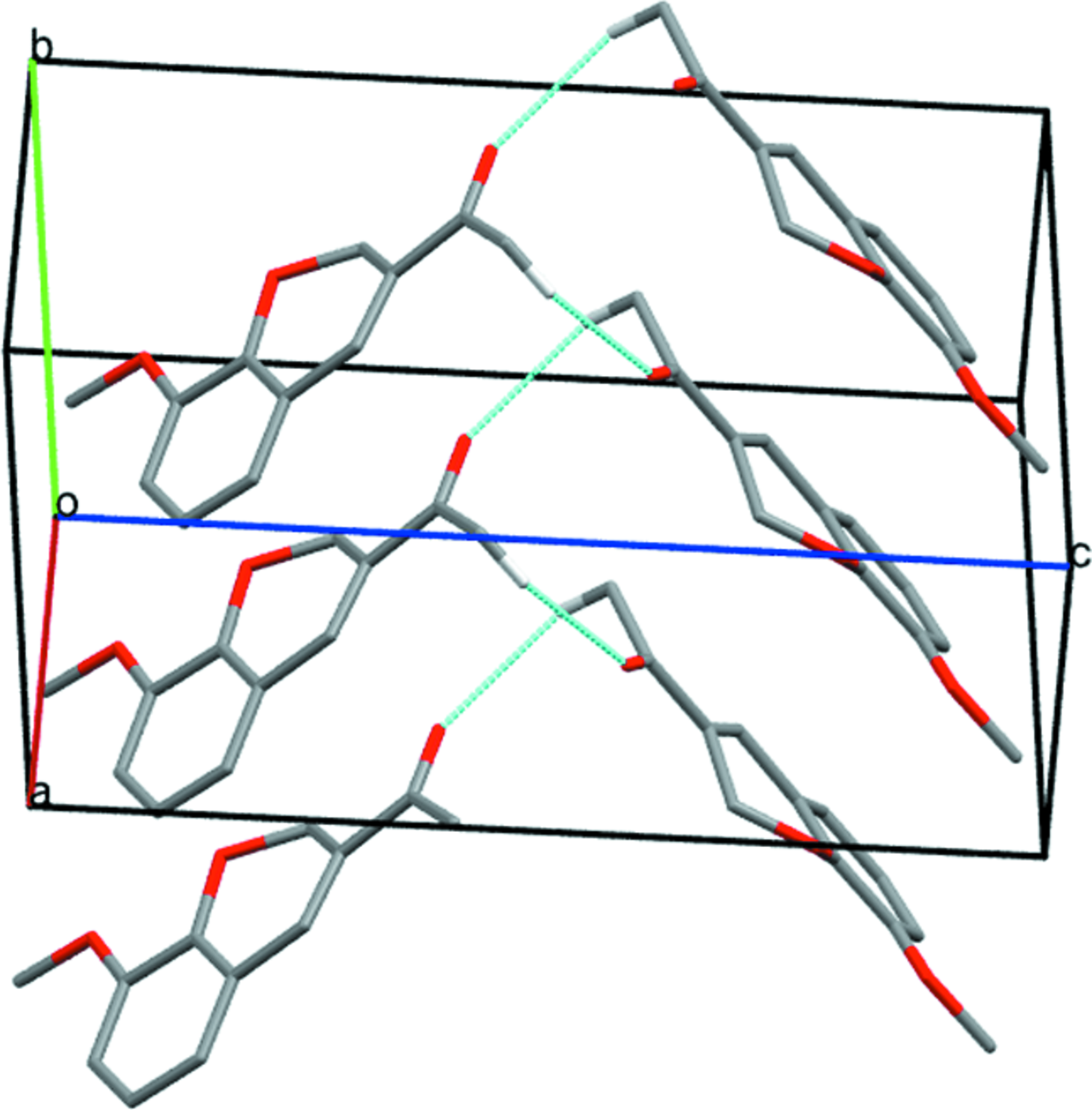

Supplement: Supplementary file 5 [file e-70-0o936-fig2.tif]
